# An Exploration of Dietary Strategies for Hypertension Management: A Narrative Review

**DOI:** 10.7759/cureus.50130

**Published:** 2023-12-07

**Authors:** Abdullh A Altawili, Mohammed Altawili, Arwa M Alwadai, Ahlam S Alahmadi, Abdulrahman Mohammed A Alshehri, Badriah H Muyini, Abdullah R Alshwwaf, Abdullah M Almarzooq, Abdulaziz Hassan A Alqarni, Zain Amer L Alruwili, Mawadah M Alharbi, Yahya M Alrashed, Nashi M Almuhanna

**Affiliations:** 1 Internal Medicine and Gastroenterology, King Fahad Specialist Hospital, Tabuk, SAU; 2 General Practice, Al Aziziyah Primary Health Care Center, Tabuk, SAU; 3 Internal Medicine, Najran University, Najran, SAU; 4 Internal Medicine, AlMaarefa University, Riyadh, SAU; 5 General Practice, Khamis Mushayt General Hospital, Abha, SAU; 6 Internal Medicine, King Khalid University, Abha, SAU; 7 General Practice, Aloyun Hospital, Alahsaa, SAU; 8 General Practice, Khafji General Hospital, Khafji, SAU; 9 General Practice, Khamis Mushayt General Hospital, Khamis Mushait, SAU; 10 Internal Medicine, Northern Border University, Arar, SAU; 11 Internal Medicine, Taibah University, Medina, SAU; 12 Medical-Surgical Nursing, Prince Sultan Military Medical City, Riyadh, SAU

**Keywords:** plant-based diet, low-sodium diet, dietary fiber, vegetarian diet, dash diet, mediterranean diet, anti-hypertensive diet, dietary strategies, nutrition, hypertension

## Abstract

This review aims to clarify the influence of various nutritional approaches in the management of hypertension. An extensive search of databases, namely, PubMed, Cochrane Library, Scopus, and Web of Science, was performed, covering the period from January 2012 to October 2023. We used keywords, such as "hypertension," "nutrition," "nutritional management," "nutrient intake," "dietary strategies," "DASH diet," "Mediterranean diet," and related terms. Hypertension, a grave global health concern, affects more than one billion people worldwide. Lifestyle modifications, including nutritional strategies, are important in non-pharmacological interventions. Dietary patterns, such as the DASH (Dietary Approaches to Stop Hypertension) and Mediterranean diets, which emphasize fruit and vegetable consumption, have demonstrated efficacy in reducing blood pressure. Certain nutrients, such as potassium, calcium, and magnesium, have significant effects on blood pressure. The mechanisms underlying these dietary strategies include vasodilation, improvement in endothelial function, regulation of sodium balance, and mitigation of oxidative stress. However, successful implementation of these strategies can be hindered by various factors, such as adherence challenges, socioeconomic disparities, and cultural preferences. In conclusion, robust evidence supports the effectiveness of nutritional strategies in the management of hypertension. DASH and Mediterranean diets, along with an increased intake of specific nutrients, contribute to blood pressure reduction. Hence, comprehensive lifestyle modifications, with a focus on nutritional changes, are recommended as primary or complementary treatments for hypertension. Addressing the identified barriers could enhance the efficacy and use of these dietary approaches in clinical practice.

## Introduction and background

Hypertension, also known as the silent killer, is a widespread health condition affecting over a billion people across the globe, with considerable burden on the healthcare system [1]. It is estimated to affect around a third of the adult population worldwide, with projections suggesting an increase of up to 20% by 2025 [1]. It is recognized as a primary risk factor for various cardiovascular conditions, such as stroke, heart failure, and coronary artery disease, and it also significantly contributes to chronic kidney disease [2]. The pathophysiological complexities of hypertension are manifold, intertwining physiological systems and molecular pathways. Consequently, the persistent exploration and management of hypertension remain crucial in both biomedical research and clinical practice. Traditionally, the primary approach to managing hypertension is a pharmaceutical intervention. However, in recent years, there has been a paradigm shift toward incorporating lifestyle modifications, with a particular emphasis on nutritional strategies, as a part of a comprehensive approach to managing this condition [3].

Recent studies have begun to highlight the influential role of dietary and nutritional strategies in preventing and managing hypertension [3]. There is growing evidence suggesting that diet can control blood pressure levels, both directly and indirectly, with certain dietary patterns and nutrients potentially having antihypertensive effects [4]. Five dietary approaches are associated with lower blood pressure. However, DASH (Dietary Approaches to Stop Hypertension) diet and the Mediterranean diet have received extensive scientific scrutiny and are recommended by health organizations because of their potential to reduce blood pressure [5]. These diets are characterized by a high intake of fruits, vegetables, whole grains, lean proteins, and low-fat dairy products, and they minimize the consumption of sodium, saturated fats, and added sugars [5]. The impact of specific nutrients, such as potassium, magnesium, and calcium, in addition to dietary fiber and certain plant-derived phenolic compounds, on hypertension management has also been a subject of investigation [6,7]. Nevertheless, the precise mechanisms by which these dietary strategies achieve their antihypertensive effects remain elusive.

Although the direct impacts on blood pressure regulation are well established, the associated molecular, metabolic, and physiological mechanisms necessitate additional research [6,7]. The real-world effectiveness of these nutritional strategies is subject to various influences, including patient compliance, cultural dietary preferences, socioeconomic circumstances, and the level of support provided by healthcare practitioners [8]. Therefore, understanding these influences and developing strategies to overcome potential obstacles is the goal. Healthcare professionals, especially physicians, play a crucial role in patient education regarding the significance of nutritional strategies in managing blood pressure. Physicians’ advice is the primary source of reliable health information for most patients and can profoundly influence patients’ dietary habits [9]. Although numerous studies have been conducted to establish the efficacy of these nutritional strategies, there remains a need for comprehensive and narrative reviews that can clarify these findings for clinical practice.

This review offers outstanding evidence of the role of nutritional strategies in hypertensive management. This study discusses various dietary approaches, the significance and mechanisms of these strategies, the roles of different nutrients and food groups, and the challenges in implementing these strategies. Furthermore, the review underlines the crucial role of healthcare providers in patient education and offers future research recommendations.

## Review

Materials and methods

We conducted an extensive literature search to identify studies that evaluated the impact of nutritional strategies on hypertension with clinical improvement. Four primary electronic databases, namely, PubMed, the Cochrane Library, Scopus, and Web of Science, were systematically searched from January 2012 to October 2023. Search terms, such as "hypertension," "high blood pressure," "nutrition," "nutritional management," "nutrient intake," "dietary strategies," "dietary approaches," "dietary patterns," "anti-hypertensive diet," "DASH diet," "Mediterranean diet," "dietary fiber," "low-sodium diet," "plant-based diet," "vegetarian diet," "potassium," "magnesium," "calcium," "beetroot," "garlic," "flaxseeds," "blood pressure control," "lifestyle modifications," "cardiovascular disease," "prevention," and "nutraceuticals," were used. We considered a selection of systematic reviews, meta-analyses, interventional studies, cohort studies, and epidemiological studies, all published in English, focusing on the impact of nutrition on blood pressure control.

Hypertension: burden, pathophysiology, and management

Hypertension is a crucial challenge to the worldwide healthcare system. Globally, it is estimated that there are 1.28 billion adults between the ages of 30 and 79 years who have hypertension. Most of these individuals, approximately two-thirds, are from low- and middle-income countries [1]. The awareness of hypertension among adults is relatively low, with an estimated 46% of adults with the condition not realizing that they have it. Moreover, less than half (42%) of adults with hypertension are being treated [1]. Among those being treated, only about one in five (21%) have the condition under control [1]. Hypertension is a leading cause of premature death worldwide. It is a part of the global targets for non-communicable diseases to reduce the prevalence of hypertension by a significant 33% between 2010 and 2030 [1]. The prevalence of hypertension varies across regions and income groups. The WHO African Region has the highest prevalence (27%), whereas the WHO Region of the America has the lowest prevalence (18%). The number of adults with hypertension has drastically increased from 594 million in 1975 to 1.13 billion in 2015, with the increase mainly seen in low- and middle-income countries [1]. This increase is attributed primarily to an increase in the risk factors for hypertension in these populations [1].

The WHO is actively involved in supporting countries in reducing the public health burden of hypertension. Since 2017, through the implementation of the Global Hearts Initiative in 31 low- and middle-income countries, 7.5 million people have received protocol-based hypertension treatment through person-centred models of care, demonstrating the feasibility and effectiveness of standardized hypertension control programs [1].

The pathophysiology of hypertension is multifactorial and involves complicated interactions between genetic predisposition and environmental triggers [10]. Two primary mechanisms that contribute to pathophysiology are an increase in peripheral resistance, primarily due to functional and structural changes in the peripheral arterioles, and an increase in cardiac output to overcome high afterload. These mechanisms are triggered by various factors, including the nervous system, renal system, and hormonal regulation. For instance, overactivity of the sympathetic nervous system stimulates the renin-angiotensin-aldosterone system (RAAS) [10]. This results in vasoconstriction, sodium-water retention, and fluid overload, leading to increased blood pressure. In addition, the development of hypertension has been significantly attributed to endothelial dysfunction, which is distinguished by a decline in the bioavailability of nitric oxide, an essential vasodilator. Furthermore, insulin resistance and chronic persistent hyperinsulinemia may play a role in pathophysiology by promoting sodium retention and stimulating the sympathetic nervous system [10].

The management of hypertension requires a comprehensive approach that integrates lifestyle modifications and pharmacotherapy. Lifestyle interventions include a healthy diet, such as the DASH diet, physical activity, reduction of alcohol consumption, smoking cessation, and stress management [1-4]. These non-pharmacological interventions can substantially reduce blood pressure because they are essential components of hypertension pathogenesis [1-4]. The therapeutic strategy for hypertension strives to decrease arterial pressure elevations and minimize the probability of cardiovascular pathologies. The 2023 guidelines issued by the European Society of Hypertension advocate the use of five primary categories of pharmaceutical agents: angiotensin-converting enzyme inhibitors, angiotensin receptor blockers, calcium channel blockers, thiazide or thiazide-like diuretics, and beta-blockers [3]. Among these, the use of a renin-angiotensin system inhibitor is preferred, barring contraindications, as its effectiveness in reducing both blood pressure and adverse cardiovascular outcomes has been corroborated by multiple randomized controlled trials [2,3]. The guidelines further recommend the initiation of a combined therapy involving two drugs encapsulated in a single pill, emphasizing its superior efficacy in controlling blood pressure compared with monotherapy [3]. Non-pharmacological interventions involving lifestyle modifications, such as weight management, sodium intake reduction, increased potassium consumption, regular physical exercise, limited alcohol consumption, and smoking cessation, are also recommended for all hypertensive patients [2,3]. Overall, these guidelines provide evidence-based recommendations for both non-pharmacological and pharmacological management of hypertension, aiming for effective blood pressure control and consequent reduction in cardiovascular risk [3].

Different nutritional/dietary strategies 

The management of hypertension extends beyond pharmacological intervention, with dietary modification serving as a key component of non-pharmacological management [1]. Five diet strategies have been identified as effective: the DASH diet, Mediterranean diet, low-sodium diet, vegetarian diet, and portfolio diet (Table 1). However, it is crucial to tailor these dietary changes to each patient’s nutritional needs and preferences to ensure long-term adherence and effectiveness [2,3]. First, the DASH diet emphasizes fruits, vegetables, whole grains, lean proteins, and low-fat dairy products while limiting saturated fats, cholesterol, and sodium. Randomized controlled trials have demonstrated a significant reduction in systolic and diastolic blood pressure by 11.4 and 5.5 mmHg, respectively, in patients following the DASH diet [4,5]. Second, the Mediterranean diet, characterized by a high intake of fruits, vegetables, whole grains, olive oil, and lean proteins, such as fish and poultry, has been associated with improved blood pressure control. Its emphasis on monounsaturated fats and polyphenols contributes to its antihypertensive effect, with a reported 7.1 mmHg reduction in systolic blood pressure in a recent meta-analysis [11]. Third, overconsumption of sodium can induce water retention, thereby escalating blood pressure. A prospective study showed that adopting a low-sodium diet, often supplemented with increased potassium, resulted in an average 4.8 mmHg decrease in systolic blood pressure [12]. Fourth, a vegetarian diet, which eliminates meat and sometimes other animal products, has been associated with lower blood pressure levels. The high intake of fiber, potassium, and antioxidants, along with lower saturated fat and cholesterol intake, is believed to contribute to this effect, with studies showing a 5-7 mmHg reduction in systolic blood pressure [13]. Finally, the portfolio diet, which includes a mix of plant sterols, soy protein, viscous fibers, and nuts, has also shown promise in managing hypertension. This diet is rich in compounds associated with lower blood pressure, such as potassium, magnesium, fiber, and plant proteins. A recent study showed an average decrease of 5.2 mmHg in systolic blood pressure among those adhering to the portfolio diet [14].

**Table 1:** Comparison of different diets for hypertension management.

Diet	Key components	Benefits	Potential challenges
Dietary Approaches to Stop Hypertension (DASH) diet	Rich in fruits, vegetables, whole grains, and low-fat dairy products. Includes meat, fish, poultry, nuts, and beans. Limited in sugar-sweetened foods and beverages, red meat, and added fats.	Proven to lower blood pressure and is also linked to lower cholesterol levels.	May require significant changes from a person's current diet.
Mediterranean diet	High in fruits, vegetables, whole grains, beans, nuts and seeds, and olive oil. Includes moderate amounts of dairy products, poultry, and fish. Red meat and sweets are eaten sparingly.	Associated with reduced risk of cardiovascular diseases, including hypertension. Also linked to improved overall health and well-being.	May be more expensive due to high intake of fruits, vegetables, and fish. Requires cooking and preparation time.
Low-sodium diet	Restricts the intake of sodium to 1,500 to 2,300 milligram per day.	Can directly lower blood pressure by reducing water retention.	Requires careful reading of food labels for sodium content. Eating out can be challenging due to high sodium content in restaurant foods.
Vegetarian/vegan diet	Excludes meat and animal products (vegan). Vegetarian diets may include dairy and eggs. High in fruits, vegetables, whole grains, nuts, seeds, and legumes.	Can lower blood pressure and decrease the risk of heart disease.	May require supplementation for nutrients commonly found in animal products, such as Vitamin B_12_ and iron. Requires careful meal planning to ensure adequate protein intake.
Portfolio diet	A plant-based diet that includes a mix of foods known to lower cholesterol such as nuts, soy, plant sterols, and high-fiber foods like oats and barley.	Shown to lower cholesterol levels as much as a statin in some studies. Can also lower blood pressure due to high fiber content and plant-based components.	Requires a significant shift to plant-based eating, which can be challenging for some. Portion control is important to avoid excess calorie intake.

Mechanisms beyond the antihypertensive effect of nutritional strategies

Dietary strategies play a key role in the management of hypertension, with their effects spanning various physiological processes [15]. The mechanisms through which these dietary interventions operate include the promotion of vasodilation, improvement of endothelial function, reduction of fluid retention, and mitigation of oxidative stress. Each of these mechanisms contributes significantly to the maintenance of cardiovascular health and prevention of hypertension. Vasodilation, the process of widening blood vessels, is an important factor in blood pressure regulation. Dietary strategies, such as the Mediterranean diet, which is rich in monounsaturated fats and polyphenols, enhance vasodilation. Monounsaturated fats, predominantly derived from olive oil, and polyphenols, abundant in fruits and vegetables, synergistically relax blood vessels and lower blood pressure [16]. Endothelial function, which refers to the performance of the thin layer of cells lining the interior of blood vessels, is another crucial aspect of hypertensive management.

Diets rich in fruits, vegetables, and whole grains, such as the DASH and Mediterranean diets, provide the necessary nutrients and antioxidants that improve the function of the endothelium, thus aiding in blood pressure control [15,16]. Fluid retention, often a result of excessive sodium intake, can increase blood pressure. The DASH diet and other diets that limit sodium intake address this issue by reducing sodium consumption and increasing potassium intake. Lower sodium levels decrease water retention in the body, and heightened potassium intake aids in sodium excretion, both of which help to lower blood pressure [17,18]. Oxidative stress, an imbalance between free radicals and antioxidants in the body, can cause inflammation and damage to blood vessels, which in turn increases blood pressure. Vegetarian diets, known for their high antioxidant and fiber content, along with the portfolio diet, which includes components such as plant sterols and viscous fibers, can combat oxidative stress. Antioxidants neutralize free radicals, thereby reducing inflammation and vascular damage, whereas fiber improves insulin sensitivity and promotes weight loss, both of which are associated with lower blood pressure [19,20].

Role of macronutrients in hypertension management

The management of hypertension involves a multifaceted approach that includes lifestyle changes and dietary adjustments. Each macronutrient, namely, carbohydrates, proteins, and fats, plays a distinct role in this process. Their effects on blood pressure may be more pronounced when consumed as a part of a balanced and diverse diet, such as the DASH diet or Mediterranean diet, possibly because of their combined interactions [15,16]. Carbohydrates, particularly those rich in dietary fiber, are crucial in controlling hypertension. Fiber, found in fruits, vegetables, and whole grains, contributes to feelings of fullness and assists in weight management, a key aspect of hypertensive control. A meta-analysis revealed that a high fiber intake significantly lowers both systolic and diastolic blood pressure [21]. Moreover, fiber can help regulate blood glucose levels, thereby reducing the risk of insulin resistance, which is often correlated with hypertension [22]. Dietary proteins also contribute to blood pressure regulation. A systematic review showed that increased dietary protein intake from both animal and plant sources leads to reductions in systolic and diastolic blood pressure [23]. Certain amino acids, notably L-arginine present in protein-rich foods, may increase the production of nitric oxide, a molecule that helps dilate blood vessels, thus lowering blood pressure [24]. Regarding dietary fats, their impact on hypertension is determined by the type of fat consumed. Monounsaturated and polyunsaturated fats, found in foods such as olive oil, nuts, seeds, fish, and avocados, have been associated with lower blood pressure [25]. Conversely, saturated and trans fats, which are often found in processed foods and animal products, have been linked to higher blood pressure [26]. These fats can negatively affect blood lipid levels, thereby possibly elevating blood pressure [27].

Role of micronutrients in hypertensive management

Micronutrients, including vitamins and minerals, play a vital role in hypertensive management. Evidence from numerous studies shows that adequate intake of certain micronutrients can help regulate blood pressure levels [28-30] (Table 2). Extensive research has reinforced the role of potassium, an indispensable mineral, in the management of blood pressure. Aburto et al. (2013) conducted a meta-analysis that identified a significant inverse correlation between dietary potassium intake and blood pressure levels in adults. This relationship was statistically significant with a correlation coefficient (*r*) of -0.13 for systolic blood pressure and -0.11 for diastolic blood pressure (P<0.05 for both), suggesting that an increase in dietary potassium intake could lower blood pressure, particularly in individuals with high sodium intake [28]. Calcium and magnesium are two additional essential minerals that have been linked to the management of hypertension. Kass et al. (2012) conducted a systematic review involving 22 trials with 1,173 participants. They found that magnesium supplementation resulted in a mean reduction of 4.18 mmHg in systolic blood pressure and 2.27 mmHg in diastolic blood pressure (P<0.001 for both) [29]. By contrast, Cormick et al. (2020), in a Cochrane review, analyzed data from 13 trials with 15,730 pregnant women. Their findings demonstrated that calcium supplementation significantly reduced the risk of high blood pressure complications in pregnancy, with a relative risk (RR) of 0.45 for gestational hypertension (95% confidence interval (CI) 0.31-0.65) and an RR of 0.35 for pre-eclampsia (95% CI 0.24 to 0.52), indicating that calcium supplementation can significantly lower the risk of hypertensive disorders during pregnancy [30].

**Table 2:** Recommended daily nutrient intake for hypertension management.

Nutrient	Recommended daily intake	Potential benefits
Sodium	Less than 2,300 milligrams or ideally 1,500 milligrams for people with hypertension	Lower sodium intake can help reduce blood pressure levels. High sodium consumption is linked to increased blood pressure.
Potassium	3,500-4,700 milligrams	Potassium can help balance the amount of sodium in cells, which can aid in blood pressure control.
Magnesium	310-420 milligrams for adults	Magnesium can help lower blood pressure levels. It also aids in nerve function, blood glucose control, and protein synthesis.
Calcium	1000-1300 milligrams for adults	Calcium can help blood vessels tighten and loosen, thus aiding in blood pressure control. It also helps in muscle function and nerve signaling.
Fiber	25-38 grams per day	A diet high in fiber can contribute to overall heart health and indirectly aid in blood pressure control.

Vitamin D deficiency is associated with a higher risk of hypertension. In this regard, Wu et al. (2017) conducted a meta-analysis encompassing 35 randomized controlled trials with a total of 4,541 participants. Their analysis demonstrated that vitamin D supplementation led to a moderate reduction in blood pressure in patients with hypertension. Specifically, the results showed a mean decrease of 3.6 mmHg in systolic blood pressure and 1.8 mmHg in diastolic blood pressure (P<0.05 for both). This finding emphasizes the importance of maintaining appropriate vitamin D levels for managing hypertension [31]. Flavonoids, a category of plant-based micronutrients, have been examined for their ability to reduce blood pressure. In a meta-analysis conducted by Serban et al. (2016), they synthesized data from 14 trials with 594 participants. The study demonstrated that diets rich in flavonoids, especially those containing anthocyanins (a flavonoid subclass found in foods like berries and red wine), could noticeably lower blood pressure. The results showed an average decrease of 5.35 mmHg in systolic blood pressure and 3.08 mmHg in diastolic blood pressure (P<0.001 for both) [32].

There is emerging evidence on the role of vitamin C, a powerful antioxidant, in regulating blood pressure. Juraschek et al. (2012) conducted a meta-analysis by pooling data from 29 short-term trials involving 1,407 participants. Their findings indicated that vitamin C supplementation could significantly reduce both systolic and diastolic blood pressure. Specifically, the results showed a mean decrease of 3.84 mmHg for systolic blood pressure and 1.48 mmHg for diastolic blood pressure (P<0.01 for both) [33]. Furthermore, the role of micronutrients in managing hypertension may not only be due to their individual effects but also their interactions with other components of the diet [34].

Role of food groups with antihypertensive effect

The relationship between diet and hypertension is significant, with several studies illustrating the impact of certain food groups on the effective management of hypertension. Fruits and vegetables play a prominent role in this equation. A study by Bondonno et al. (2021), involving 8,663 participants, identified a direct relationship between a higher intake of these food groups and a lower risk of hypertension. The results indicated a 22% decrease in the hypertensive risk of individuals consuming more than five servings of fruits and vegetables per day [35]. Similarly, the type of meat intake is also a crucial determinant. Bechthold et al. (2019) conducted a study with 36,821 participants and demonstrated that replacing red and processed meat with lean white meat correlated with a reduced risk of hypertension. The findings showed a 19% lower risk of hypertension with high white meat consumption [36]. The benefits of whole grains are also noteworthy. A study by Reynolds et al. (2020), involving 137,130 participants from 21 countries, found a strong association between a higher intake of whole grains and a lower risk of hypertension. Those consuming at least two servings of whole grains per day exhibited a 12% lower risk of hypertension [37]. A study conducted with 4,667 participants found that a higher intake of nuts and seeds was associated with a lower risk of hypertension. For each 30g/day increase in nut intake, there was a 15% reduction in the risk of hypertension [38].

Dairy products, especially low-fat options, also play a role in the management of hypertension. Tong et al. (2022) conducted a study involving 206,000 participants and showed that a higher intake of low-fat dairy products was associated with a lower risk of developing hypertension. Their results showed a 14% reduction in hypertensive risk with daily consumption of low-fat dairy [39]. Finally, the Mediterranean diet, which is rich in fruits, vegetables, whole grains, lean meats, and low-fat dairy, was found to significantly reduce blood pressure in a study by Toledo et al. (2013) involving 7,447 participants. Their findings indicated a 20% reduction in hypertensive risk in participants strictly adhering to this diet [40]. Various food groups significantly contribute to managing hypertension, underscoring the importance of a balanced, diverse diet in maintaining optimal blood pressure levels.

Effectiveness of nutritional strategies in hypertensive management: epidemiological and interventional studies

Numerous epidemiological and interventional studies corroborate the effectiveness of dietary interventions in managing hypertension, underlining the vital role of nutrition in preventing and controlling chronic health conditions [15,16,41,42]. The influential epidemiological DASH study highlighted diet’s significant role in managing hypertension [15]. Participants following the DASH diet, which emphasizes fruits, vegetables, low-fat dairy foods, whole grains, poultry, fish, and nuts, experienced an average reduction of 5.5 mmHg in systolic blood pressure and 3.0 mmHg in diastolic blood pressure. This change was highly significant with P<0.001 [15]. The Nurses’ Health Study II, which included 88,517 women aged 34-53 years, found that higher adherence to the DASH diet was associated with a lower risk of hypertension [41]. Specifically, women in the highest quintile of the DASH score had a hazard ratio of 0.78 for developing hypertension compared with those in the lowest quintile, indicating a 22% lower risk. This association was statistically significant with a P-value <0.001 [41]. Interventional studies, such as the PREMIER clinical trial, further substantiated the role of dietary modifications in the management of hypertension [42]. The trial, which involved 810 adults with prehypertension or stage 1 hypertension, found that a comprehensive lifestyle intervention incorporating a DASH diet and increased physical activity could decrease systolic blood pressure by 4.3 mmHg and diastolic blood pressure by 2.6 mmHg. These reductions were statistically significant with P<0.001 [42]. The PREDIMED study investigated the effects of a Mediterranean diet supplemented with extra-virgin olive oil or nuts on hypertension [16]. Participants following this diet showed a significant reduction in blood pressure. There was a decrease in systolic and diastolic blood pressure by 5.9 and 3.8 mmHg, respectively, with a P-value <0.01 [16].

Challenges in implementing nutritional strategies

Implementing nutritional strategies for hypertension management often comes with a set of challenges. Although nutritional strategies can be effective in managing hypertension, several factors, such as adherence, socioeconomic status, and cultural preferences, can pose significant challenges to their implementation. A significant challenge is adherence to dietary changes. In one study, out of 810 adults with prehypertension or stage 1 hypertension, only approximately 35% of the participants managed to maintain the recommended dietary patterns over the course of 18 months [43]. Moreover, the reduction in sodium intake, which is crucial in managing hypertension, was difficult to maintain in the long term [43]. Another study reported that adherence to a low-sodium diet was challenging because of the ubiquity of sodium in processed and restaurant foods [44].

Several studies have indicated that lower socioeconomic status is associated with a poor-quality diet that is high in sodium and low in fruits, vegetables, and whole grains, which are essential elements of a heart-healthy diet [45]. A large-scale observational study with 102,216 participants found that lower socioeconomic status is associated with a 17% increased risk of developing hypertension [46]. Furthermore, a systematic review and meta-analysis of 42 studies concluded that socioeconomic disparities in hypertension could be attributed to differences in dietary patterns [47]. Cultural preferences can pose significant challenges. A study involving 1681 patients with hypertension found that culturally tailored dietary advice was more effective than generic dietary advice [48]. This suggests that dietary habits deeply rooted in cultural practices can be a barrier to the implementation of nutritional strategies for hypertension management [48]. Another study involving African Americans, a population with a high prevalence of hypertension, demonstrated that a culturally tailored education program improved adherence to the DASH diet and reduced blood pressure [49].

Role of physicians and healthcare providers in patient education

The role of physicians and other healthcare providers in patient education regarding the benefits of nutritional interventions for hypertension management is crucial. The American Heart Association (AHA) recommends that physicians provide dietary advice or refer patients to a dietitian. This recommendation is supported by several studies that have suggested that physicians’ advice strongly influences patients’ dietary behaviors [49]. In a study, the implementation of the DASH diet resulted in significant reductions in systolic and diastolic blood pressure. In this randomized controlled trial involving 459 adults, the DASH diet was shown to reduce systolic blood pressure by an average of 11.4 mmHg and diastolic blood pressure by 5.5 mmHg compared with the control diet [15]. A systematic review and meta-analysis found that dietary nitrate supplementation, mainly from beetroot juice, led to significant reductions in blood pressure in hypertensive individuals [50]. These findings suggest that physicians and other healthcare providers can educate patients about the potential benefits of dietary nitrate supplementation as a part of a comprehensive hypertensive management plan. A long-term study found that a Mediterranean diet supplemented with extra-virgin olive oil or nuts significantly reduced the incidence of major cardiovascular events among individuals with high cardiovascular risk. These results highlight the long-term benefits of nutritional intervention in improving the quality of life and reducing cardiovascular risk, reinforcing the necessity of patient education in this regard [40]. A report from the Centers for Disease Control and Prevention (CDC) stated that nearly half of the adult population in the United States has hypertension, and only about one in four have the condition under control. This statistic indicates a significant gap in hypertensive management and underscores the urgent need for effective patient education on nutritional interventions by healthcare providers [51].

Physicians play a critical role in guiding hypertensive patients to adhere to nutritional interventions. Strategies that have shown success include education, motivation, the use of technology, and personalized advice, all of which are backed by empirical evidence and influential statistics. Education remains the cornerstone of patient adherence. A previous study demonstrated that comprehensive lifestyle modifications, including dietary changes, resulted in significant reductions in blood pressure and improved adherence to treatment [52]. In this study, a majority (69%) of participants successfully adhered to the prescribed dietary intervention, emphasizing the importance of patient education. Motivational interviewing is another effective strategy. A systematic review highlighted that this patient-centered counseling technique significantly improved lifestyle changes, including dietary modifications, among patients with chronic diseases [53]. The study found a median adherence rate of 74% among participants, indicating the effectiveness of motivational interviewing in enhancing patient commitment.

The use of technology has also been shown to significantly improve adherence. A trial revealed that a mobile app providing dietary advice improved adherence to the DASH diet, leading to substantial blood pressure reductions among hypertensive patients [54]. The study noted a 56% increase in adherence to the DASH diet among app users. Similarly, a meta-analysis showed that telemedicine interventions, such as telephone coaching and mobile apps, improved adherence to dietary interventions, resulting in significant improvements in blood pressure [55]. The study reported a 65% adherence rate in the intervention group, emphasizing the potential of digital tools in improving dietary adherence.

Finally, personalized nutrition advice based on genetic information could enhance adherence. A study suggested that personalized nutrition advice based on genetic data resulted in greater dietary behavior changes than population-based dietary advice [56]. Approximately 80% of the participants who received personalized dietary advice adhered to the recommendations, highlighting the potential of precision medicine in managing hypertension. Physicians can significantly influence hypertensive patients’ adherence to nutritional interventions through comprehensive education, motivational interviewing, digital technology, and personalized nutrition advice. As demonstrated by the high adherence rates in the cited studies, these strategies can lead to better hypertension management and improved patient quality of life. Moreover, Aldana et al. declared that the Ornish diet appears to cause improvements in cardiovascular risk factors with a 44% reduction rate in anginal events after 12 months [57].

Future research

The role of nutrition in hypertensive management is a field ripe for further exploration. Despite significant progress, gaps remain in the literature that future research should address. Among these is the need for a deeper understanding of the effects of comprehensive dietary patterns on hypertension. While the impact of individual nutrients has been well studied, the influence exerted by combinations of foods and nutrients is less clear. Moreover, most current dietary guidelines are generalized, often failing to account for individual variations. Research into personalized nutrition strategies, considering factors, such as genetics, age, sex, and lifestyle, could lead to more tailored and, consequently, more effective hypertension management. A common stumbling block in nutritional interventions is long-term adherence. Therefore, future studies exploring strategies to encourage lasting dietary changes are invaluable. This might involve delving into the realm of behavioral interventions, leveraging technology to support dietary changes, or examining the role of community and family support in fostering adherence.

Another potentially fruitful area of investigation is nutrient bioavailability. Understanding how factors, such as food preparation or the simultaneous consumption of other foods, affect the proportion of nutrients that are absorbed and used by the body can enhance the effectiveness of nutritional strategies for hypertension. In addition, there is a pressing need for research aimed at understanding the most effective ways to educate patients about the crucial role of nutrition in hypertensive management. This could encompass the evaluation of various educational strategies, tools, and resources, as well as the exploration of the role of different healthcare professionals in providing this education.

Finally, much of the current research focuses on populations with relatively easy access to a wide variety of foods. Future research should strive to include underserved populations, who may face unique challenges, such as limited availability of healthy foods or economic barriers to accessing them. By addressing these gaps in our understanding, future research can pave the way for more effective, personalized, and sustainable nutritional strategies for the management of hypertension.

## Conclusions

Hypertension presents a complex health challenge, and nutrition has emerged as a central component in its management. The contribution of various macronutrients, micronutrients, and specific food groups to the modulation of blood pressure levels is well established. Empirical evidence from numerous epidemiological and interventional studies underscores the powerful potential of nutritional strategies in combating hypertension. However, the practical implementation of these strategies is fraught with barriers. These include the need for effective patient education, motivation, and long-term adherence to recommended dietary changes. In this context, healthcare providers, particularly physicians, play a fundamental role. Their contribution to enhancing patient understanding and ensuring adherence to nutritional guidance is invaluable.

In the future, we anticipate further refinement of dietary strategies, innovative approaches to overcoming implementation challenges, and more effective use of healthcare providers’ role in patient education. Managing hypertension is a complex task that necessitates a comprehensive approach. Nutrition, with its extensive preventive and therapeutic potential, remains an essential component of this strategy. As our understanding deepens, we are inching closer to a future where hypertension can be effectively controlled through personalized, scientifically backed nutritional strategies.
